# Molecular characterization, function, tissue differential expression, and single-nucleotide polymorphism of buffalo *TP53* gene

**DOI:** 10.5194/aab-67-217-2024

**Published:** 2024-05-21

**Authors:** Lige Huang, Dan Sheng, Xinyang Fan, Ruixia Gao, Yongwang Miao

**Affiliations:** 1 Faculty of Animal Science and Technology, Yunnan Agricultural University, Kunming, Yunnan, China

## Abstract

*TP53* has been shown to be involved in lactation in cattle. However, the role of *TP53* in buffalo lactation remains unknown. To this end, we isolated and identified the complete coding sequence (CDS) of the *TP53* gene from the buffalo mammary gland and further analyzed its molecular characteristics, function, tissue differential expression, and single-nucleotide polymorphism (SNP). A transcript of this gene was cloned with a CDS length of 1161 bp, encoding a protein consisting of 386 amino acid residues. Bioinformatics analysis showed that buffalo *TP53* CDS and the physicochemical characteristics, conserved domains, structure, and function of its encoded protein are highly similar to those of other species in Bovidae. The buffalo TP53 protein contains an N-terminal activation domain, a DNA-binding domain, and a tetrameric domain, and it plays a functional role in the nucleus. *TP53* was found to express in all 11 detected buffalo tissues, and its expression in the heart, kidney, brain, muscle, and rumen during lactation was significantly higher than that during non-lactation ( p<0.05 ), while in the liver, lung, and mammary gland, its expression was the opposite ( p<0.05 ). Interference experiments in buffalo mammary epithelial cells (BuMECs) showed that *TP53* inhibits the expression of genes related to milk protein and milk fat synthesis through the PI3K–AKT–mTOR pathway. A synonymous nucleotide substitution (c.204C > T) was found in the *TP53* CDS of river buffalo, which is the CC homozygote in swamp buffalo. The results indicate that the *TP53* gene is involved in buffalo lactation by negatively regulating the synthesis of milk protein and milk fat.

## 1 Introduction

The *TP53* (tumor protein 53, TP53/P53) gene is the tumor suppressor gene with the highest mutation frequency in tumorgenesis, and about 50 % of human cancers are associated with *TP53* mutations (Vogelstein et al., 2000). As a transcription factor, TP53 trans-activates the transcription of downstream target genes by specifically binding to their regulatory sequences in response to a variety of cellular stresses, thus participating in a variety of biological processes such as cell cycle control, apoptosis, aging, differentiation, and DNA repair (Levine and Oren, 2009). In addition, the activation of TP53 in human lung cancer cells leads to dephosphorylation of the eukaryotic translation initiation factor 4E-binding protein 1 (4EBP1), which increases the binding of eukaryotic initiation factor 4E (EIF4E) to 4EBP1 and then inhibits protein synthesis (Tilleray et al., 2006). In mouse erythroleukemia cells, the activation of TP53 decreased the phosphorylation of 4EBP1 and the activity of ribosomal protein S6 kinase (S6k), resulting in downregulation of protein synthesis (Horton et al., 2002). With further research, TP53 has been revealed to be involved in cellular glycolysis, oxidative phosphorylation, amino acid metabolism, and other metabolic pathways (Maddocks and Vousden, 2011). Subsequently, TP53 has been proven to be involved in regulating the expression of genes related to lipid metabolism, thereby promoting fatty acid oxidation and inhibiting lipid synthesis (Goldstein and Rotter, 2012). In mouse 3T3-L1 preadipocytes, the knockout of the *TP53* gene increases fat synthesis and adipogenic gene expression, while its overexpression results in the opposite (Okita et al., 2014). The TP53 signaling pathway is also significantly correlated with the milk yield, protein yield, and lactose rate of Holstein cows (Ammah et al., 2018).

The buffalo *TP53* gene is located on chromosome 3, and the NCBI gene database (https://www.ncbi.nlm.nih.gov/, last access: 4 February 2024) shows that there are three transcript variants in the mRNA sequences of the buffalo gene. The transcript variant X1 (NM_001290844) contains 10 exons, with a complete coding sequence (CDS) of 1161 bp in length, which encodes a polypeptide containing 386 amino acid residues, while transcript variants X2 (XM_006062871) and X3 (XM_044938378) were predicted from the buffalo genomic data, and both of them contain 11 exons (https://www.ncbi.nlm.nih.gov/, last access: 4 February 2024). It has been determined that buffalo TP53 protein plays a functional role in the nucleus (Singh et al., 2015). In addition, buffalo TP53 protein was found to efficiently transcribe and activate the cyclin-dependent kinase inhibitor 1A (CDKN1A) in buffalo (Singh et al., 2015). In cattle, the *TP53* gene is located on chromosome 19, containing 11 exons and 10 introns, and its CDS (NM_174201) length is also 1161 bp (https://www.ncbi.nlm.nih.gov/, last access: 4 February 2024).

Buffalo are divided into two distinct subtypes: river buffalo ( 2n=50 ) and swamp buffalo ( 2n=48 ) (Shi et al., 2012). River buffalo are used primarily for milk production, and swamp buffalo are primarily used for draft. Buffalo milk is the second largest source of milk around the world, accounting for 13 % of the world's total milk production (Nasr et al., 2016). China is the third largest country in the world with respect to buffalo farming, after India and Pakistan (Abdel Hamid et al., 2023), and the Binglangjiang buffalo is a river-type buffalo that has been found in western Yunnan, China, in recent years. Compared with cow's milk, buffalo milk is rich in protein (especially casein), fat, and lactose as well as minerals, such as calcium, magnesium, and inorganic phosphate (Ahmad et al., 2008). So far, it has been found that TP53 plays an important role in the metabolic regulation of some mammals, but its functional role in buffalo, particularly in lactation, remains unclear. The aim of this study is to isolate and identify the buffalo *TP53* gene from the tissue of the buffalo mammary gland and to further describe its molecular characteristics, function, tissue differential expression, interaction, and single-nucleotide polymorphism (SNP) through bioinformatics, comparative genomic methods, and gene function experiments.

## 2 Materials and methods

### 2.1 Sample collection

Six Binglangjiang buffalo with their third calf aged about 4 years were selected for sample collection, including three in the peak lactation period (about 60 d postpartum) and three in the dry lactation period (about 60 d before parturition). All the buffalo sampled were fed and managed under the same conditions. After the buffalo were slaughtered, the tissue samples used to extract total RNA were collected immediately from the heart, liver, spleen, lung, kidney, brain, cerebellum, small intestine, muscle, mammary gland, and rumen, and then we put them into RNase-free freezing tubes, stored them in liquid nitrogen, and then took them back to the laboratory for total RNA preparation.

The blood samples used for SNP detection of *TP53* CDS included those of 60 Binglangjiang buffalo (river type) and those of 60 Dehong buffalo (swamp type). The buffalo were all adults and were not directly related. The Binglangjiang buffalo and Dehong buffalo were sampled from their core distribution areas, Tengchong and Mangshi in Yunnan Province, China, respectively. Approximately 4 mL of blood sample was collected from each buffalo in centrifuge tubes containing EDTA anticoagulant and were stored in the laboratory at low temperatures for DNA extraction.

### 2.2 Isolation and identification of *TP53*

Total RNA was extracted using the RNAiso Plus kit (TaKaRa, Dalian, China) according to the merchant product manual. The concentration and purity of RNA were detected by an ultraviolet spectrophotometer (Thermo Fisher Scientific, CA, USA), and then the integrity of the RNA was assessed through 1 % agarose gel electrophoresis. The cDNA was synthesized from 2–3 µ g RNA using the Reverse Transcription Kit (TaKaRa, Dalian, China) and Oligo(dT) ${}_{18}$ (50 µ M; TaKaRa, Dalian, China) as the primer. Then, the cDNA was diluted to 100 ng mL ${}^{-1}$ and kept in a refrigerator at -20 °C.

Primers for isolating the CDS of buffalo *TP53* were designed using the mRNA sequence (XM_006062871) of buffalo *TP53* as the template (Table S1 in the Supplement). The total volume of the PCR reaction system was 10 µ L, which contained 5 µ L of 2× Es Taq Master Mix (CWBIO, Beijing, China), 0.5 µ L (each) of forward and reverse primers (10 µ mol L ${}^{-1}$ ), 1 µ L of the template (100 ng µ L ${}^{-1}$ ), and 3 µ L of ddH ${}_{2}$ O. The PCR was conducted under pre-denaturation at 94 °C for 3 min, followed by 35 cycles of denaturation at 94 °C for 30 s, annealing at 63.9 °C for 40 s, extension at 72 °C for 30 s, and a final extension at 72 °C for 5 min, and then it was stopped at 4 °C. PCR products were detected by 1 % agarose gel electrophoresis, and the target band was cut and further purified using the TIANgel Purification Kit (TIANGEN, Beijing, China). The purified PCR products were linked to the pMD-18T vector (TaKaRa, Dalian, China) and performed molecular cloning. A total of 60 clones were selected and sequenced bidirectionally.

The obtained sequence of buffalo *TP53* was checked and proofread using the Lasergene software package (DNASTAR Inc.), and the open reading frame (ORF) was identified by ORF Finder (https://www.ncbi.nlm.nih.gov/orffinder/, last access: 5 March 2023). In order to confirm that the obtained sequence is the target sequence, a homologous search was conducted in the NCBI database, using it as the query sequence, by means of the online BLAST program (https://blast.ncbi.nlm.nih.gov/Blast.cgi, last access: 5 March 2023).

### 2.3 Molecular characteristics and function prediction

TP53 sequences of 13 species were recruited from the NCBI database for the analysis of sequence identity, gene structure, motif, and conserved domain (Table S2). The *TP53* structure information of the species of Bovidae, human, rat, horse, deer, camel, and pig was derived from GTF files downloaded from the NCBI database (https://www.ncbi.nlm.nih.gov/datasets/, last access: 6 March 2023). The files of each species were further processed by TBtools, and the transcriptional region structure of *TP53*, including untranslated regions and exon–intron structure for each species, was visualized by the online Gene Structure Display Server (http://gsds.gao-lab.org/, last access: 6 March 2023). The nucleotide sequence consistency among multiple species was estimated by MegAlign (DNASTAR Inc.). Based on Jones–Taylor–Thornton and gamma-distributed models, the phylogenetic analysis of TP53 protein sequences was constructed using MEGA7 (Kumar et al., 2016). The motif composition for TP53 was obtained by submitting its amino acid sequences to the MEME Suite website (https://meme-suite.org/meme/, last access: 6 March 2023), and the conserved structural domain was predicted by submitting its protein sequences to the Conserved Domain Database (https://www.ncbi.nlm.nih.gov/cdd/?term=, last access: 6 March 2023) in the NCBI. The functional modification site, physical and chemical characteristics, hydrophilicity, signal peptide, and transmembrane domain of TP53 were predicted using the online software PROSITE (http://prosite.expasy.org/prosite.html, last access: 6 March 2023), ProtParam (https://web.expasy.org/protparam/, last access: 6 March 2023), ProtScale (https://web.expasy.org/protscale/, last access: 6 March 2023), the SignalP5.0 server (https://services.healthtech.dtu.dk/services/SignalP-5.0/, last access: 6 March 2023), and the TMHMM 2.0 server (https://services.healthtech.dtu.dk/services/TMHMM-2.0/, last access: 6 March 2023), respectively. The subcellular localization and the secondary and three-dimensional structures of the protein were determined by the online ProtComp (http://linux1.softberry.com/berry.phtml, last access: 6 March 2023), SOPMA (http://npsa-pbil.ibcp.fr/, last access: 6 March 2023), and SWISS-MODEL (http://swissmodel.expasy.org/, last access: 6 March 2023), respectively. The molecular function and biological pathway of the protein were analyzed using InterPro (http://www.ebi.ac.uk/interpro/, last access: 6 March 2023), UniProt (http://www.uniprot.org/, last access: 6 March 2023), and DAVID (http://david.ncifcrf.gov/, last access: 6 March 2023). Protein–protein interactions between the target protein and other proteins were predicted by the online STRING (https://string-db.org/, last access: 6 March 2023).

### 2.4 Cell culture

Buffalo mammary epithelial cells (BuMECs) were obtained from the mammary tissue of lactating buffalo (60 d postpartum), as previously described in our laboratory (Fan et al., 2020). Purified and identified BuMECs were cultured in Dulbecco's modified Eagle medium (DMEM) containing 5 µ g mL ${}^{-1}$ hydrocortisone (Sigma, St. Louis, MO, USA), 5 µ g mL ${}^{-1}$ insulin (Sigma, St. Louis, MO, USA), 1 µ g mL ${}^{-1}$ epidermal growth factor (Sigma, St. Louis, MO, USA), 2 % penicillin–streptomycin (Gibco, Carlsbad, CA, USA), and 10 % fetal bovine serum (Gibco, Carlsbad, CA, USA) and were incubated under the conditions of 37 °C and 5 % CO ${}_{2}$ . The medium was changed every 48 h. Before the beginning of experiments, BuMECs were cultured for 48 h in the above medium with 3 µ g mL ${}^{-1}$ prolactin (Sigma, St. Louis, MO, USA) to induce lactogenesis. Finally, the medium was replaced with hormone- and growth-factor-free DMEM supplemented with 10 % fetal bovine serum.

### 2.5 Transfection of small interfering RNAs

Two specific small interfering RNAs (siRNAs) targeting different regions of *TP53* CDS (siRNA1-TP53 and siRNA2-TP53) and one non-specific negative control siRNA (NC) were designed and synthesized by Shanghai Biological Engineering Technology Services Co., Ltd. The *TP53* and NC siRNA sequences are presented in Table S1 in the Supplement. The interference efficiency of siRNA-TP53 was assessed by comparing the mRNA abundance of *TP53* in BuMECs transfected with siRNA-TP53 and transfected with NC. The expression level of *TP53* was detected by qPCR for 48 h after transfection. When the confluence of cells reached 70 %–80 %, siRNA (100 nM) with the best interference efficiency was then transfected into BuMECs using TransLipid Transfection Reagent (TransGen Biotech, Beijing, China) according to the manufacturer's instructions. Cells were harvested 48 h after transfection for RNA extraction, with three replicates for each group.

### 2.6 Detection and analysis of gene expression

The differential expression of *TP53* in lactating and non-lactating buffalo tissues and the expression of the genes related to milk protein and milk fat synthesis in BuMECs were detected by qPCR. The ubiquitously expressed housekeeping genes *ACTB*, *GAPDH*, and *RPS23* were used as internal references to normalize the results of the target gene. Primers used for qPCR detection are listed in Table S1. The qPCR was performed using the SYBR Premix Ex Taq fluorescent dye (TaKaRa, Dalian, China) and the iQ ${}_{5}$ RT-PCR instrument (Bio-Rad, CA, USA). The total volume of qPCR was 20 µ L, which contained 10 µ L of SYBR Premix Ex Taq, 0.8 µ L (each) of upstream and downstream primer (10 µ M), 6.4 µ L of ddH ${}_{2}$ O, and 2 µ L of cDNA (100 ng µ L ${}^{-1}$ ). The reaction procedure was conducted according to the manufacturer's instructions. The program LinRegPCR was used to determine the qPCR amplification efficiency of *TP53*. All analyses were performed in triplicate.

Results were statistically analyzed and visualized using GraphPad Prism 5 software (GraphPad Software Inc., La Jolla, CA, USA), and all experimental data were expressed as the means ± standard error of the means (mean ± SEM) from each of the three independent experiments. qPCR data were analyzed using the 2 ${}^{-\Delta \Delta \mathrm{Ct}}$ method relative to the control. Statistical analysis was performed using Student's t test, and either p<0.05 (significant) or p<0.01 (highly significant) was considered to be statistically significant.

### 2.7 Detection and analysis of polymorphisms

Genomic DNA was extracted from the blood sample using the method of phenol–chloroform extraction (Sambrock and Russell, 2001). The variation in the *TP53* CDS was detected by direct bidirectional sequencing of PCR products. Primers for SNP detection were designed based on the sequence of buffalo *TP53* (NC_059159; Table S1).

The PCR system contained 5 µ L of 2× Es Taq Master Mix (CWBIO, Beijing, China), 0.5 µ L (each) of upstream and downstream primers (10 µ mol L ${}^{-1}$ ), 1 µ L of DNA template (100 ng µ L ${}^{-1}$ ), and 3 µ L of ddH ${}_{2}$ O. The PCR was performed with a pre-denaturation at 94 °C for 3 min, followed by 35 cycles of denaturation at 94 °C for 30 s, annealing at a specific temperature (Table S1) for 40 s, extension for a specific time (Table S1) at 72 °C, and further extension at 72 °C for 5 min, finally stopping the reaction at 4 °C. The PCR products were detected by 1 % agarose gel electrophoresis; bands were then cut, and we purified them by means of the TIANgel Purification Kit (TIANGEN, Beijing, China). The purified PCR products were bidirectionally sequenced by Shanghai Biological Engineering Technology Services Co., Ltd (Shanghai, China).

The sequences were first checked and proofread and then were output by the SeqMan program (DNASTAR Inc.) and MEGA7 (Kumar et al., 2016) to obtain the information of SNPs. The relative synonymous codon usage (RSCU) for CDS was analyzed using the CodonW (http://codonw.sourceforge.net/, last access: 6 February 2024) program. Multiple nucleotide sequences were compared using MegAlign (DNASTAR Inc.), Clustal X (Larkin et al., 2007), and BioEdit (Hall, 1999) software, and nucleotide and amino acid difference sites were output using MEGA7 software (Kumar et al., 2016). 

## 3 Results

### 3.1 Isolation and identification of buffalo *TP53* gene

**Figure 1:**
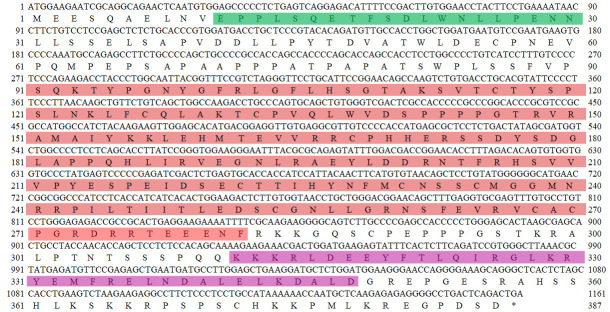
The CDS of buffalo *TP53* and its encoded amino acid sequence in this study. ATG – start codon; * – stop codon. The region in green represents the N-terminal activation domain (TAD), the region in red represents the DNA-binding domain (DBD), and the region in purple represents the tetrameric domain (TD).

In this study, only one type of transcript was obtained from the sequencing results of the 60 monoclonal colonies selected (Fig. S1 in the Supplement). The CDS of this gene was determined to be 1161 bp in length by the ORF Finder program, and the CDS was submitted to the NCBI database under the accession number of OL456217. Then the homologous search in the NCBI database using the CDS as the query sequence showed that the consistency of it with the *TP53* CDS of buffalo (NM_001290844.2), cattle (NM_174201.1), yak (XM_005894802.2), zebu (XM_019982111.1), bison (XM_010843072.1), bison (XM_010843073.1), sheep (NM_001009403.1), sheep (XM_042255225.1), goat (XM_005693530.3), and goat (XM_018064593.1) is 99.7 %, 98.3 %, 98.4 %, 98.3 %, 98.4 %, 98.3 %, 96.0 %, 93.0 %, 96.2 %, and 96.2 %, respectively (Fig. S2). Therefore, it was identified as the buffalo* TP53*. The composition of A, T, G, and C of buffalo *TP53* CDS is 23.08 %, 25.41 %, 20.67 %, and 30.84 %, respectively, and the content of G + C is 56.26 %. Buffalo *TP53 *encodes a protein composed of 386 amino acid residues (Fig. 1).

### 3.2 Structure of transcriptional region and consistency of amino acid sequence

**Figure 2:**
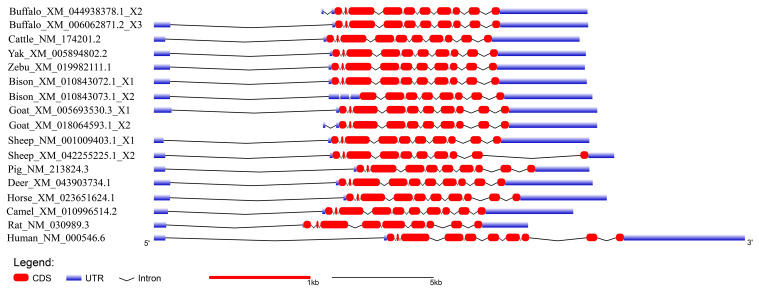
Transcriptional region structure of *TP53* in buffalo and other species.

In order to demonstrate the structural characteristics of buffalo* TP53*, the transcriptional region of this gene was compared with the homologous sequence of the livestock in Bovidae and other mammals (Fig. 2 and Table 1). Until now, there have been three buffalo *TP53 *mRNA sequences in the NCBI database, but only two of them contain flanking sequences, and they were all included in the analysis of this study. The two buffalo transcript variants have the same CDS length but differ with respect to the 5' flanking sequences. The length and structure of buffalo *TP53* CDS are nearly identical to those of cattle, yak, zebu, bison, and goat, except for the fact that the bison *TP53 *transcript variant X2 has a shorter CDS than that of buffalo, and the coding initiation site of the bison *TP53* variant X2 is located at exon 4. There are two transcriptional variants of *TP53 *in sheep, and their differences are reflected in the different positions of exon 11 in the transcriptional variants X1 and X2, which are located at positions 11 971–12 919 and 16 195–16 528 of the genome sequence (NC_056064), respectively, in accordance with the GU-AG rule for intron splicing. The sequences of the 5' flanking region and 3' flanking region for buffalo *TP53* are different from those of other species in Bovidae. The *TP53 *of all species in Bovidae contains 11 exons and 10 introns.

**Figure 3:**
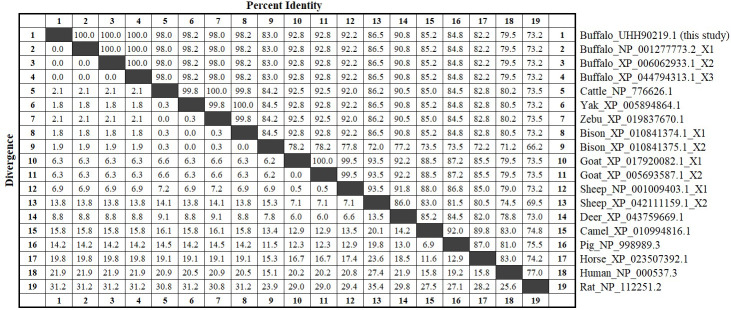
Amino acid sequence identity of TP53 between buffalo and other species. The values above the diagonals represent the consistency of the sequence, and the values below the diagonals represent the degree of divergence of the sequences.

The amino acid sequences of buffalo TP53 obtained in this study were aligned with those of some species recruited from the NCBI database (Fig. 3). The results showed that the sequence identity of TP53 from buffalo in this study is 83.0 % and 86.5 % with the TP53 transcript variant X2 from bison and sheep, respectively, and ranged from 92.2 % to 100 % with TP53 sequences from six other species of Bovidae.

**Table 1:** Structural information of *TP53* transcriptional region.

	Length (bp)													
Species	5' UTR	E1	E2	E3	E4	E5	E6	E7	E8	E9	E10	E11	3' UTR	CDS
Buffalo_X2	178	150	102	22	255	187	113	110	137	74	107	944	865	1158
Buffalo_X3	50	22	102	22	255	187	113	110	137	74	107	944	865	1158
Cattle	140	942	107	74	137	110	113	187	255	22	102	112	863	1158
Yak	186	945	107	74	137	110	113	187	255	22	101	159	866	1158
Zebu	188	944	107	74	137	110	113	187	255	22	102	160	865	1158
Bison_X1	187	159	102	22	255	187	113	110	137	74	107	942	863	1158
Bison_X2	435	159	102	84	255	187	113	110	137	74	107	947	868	972
Goat_X1	203	948	107	74	137	110	113	187	243	22	102	175	869	1146
Goat_X2	52	948	107	74	137	110	113	187	243	22	102	175	869	1146
Sheep_X1	125	949	107	74	137	110	113	187	243	22	102	97	870	1146
Sheep_X2	140	334	107	74	137	110	113	187	243	22	102	112	255	1146
Pig	140	612	107	74	137	110	113	187	255	22	102	112	533	1158
Deer	190	940	107	74	137	110	113	184	267	22	102	162	861	1167
Horse	187	930	107	74	137	110	113	187	240	22	102	159	851	1142
Camel	167	139	102	22	240	187	113	110	137	74	107	942	863	1143
Rat	131	122	83	22	273	184	223	137	74	107	531		452	1173
Human	142	1270	107	74	137	110	113	184	279	22	102	114	1191	1179

### 3.3 Physicochemical characteristics and functional modification sites

The physicochemical characteristics of buffalo TP53 were analyzed using the transcript variants of other species in Bovidae as controls, and the results showed that the basic physicochemical characteristics of buffalo TP53 are very similar to those of other species in Bovidae, except for the basic physicochemical characteristics of the TP53 transcript variant X2 in bison and sheep (Table 2). The grand average of the hydrophobicity of the buffalo TP53 protein is -0.730 , indicating that the protein is hydrophilic. Prediction showed that the buffalo TP53 protein has no signal peptide and transmembrane helix, and there are six potential functional modification sites harbored in it (Table 3).

**Table 2:** Basic physicochemical characteristics of TP53 for the species of Bovidae.

Physicochemical	Buffalo	Cattle	Yak	Zebu	Bison	Bison	Goat	Goat	Sheep	Sheep
characteristics	(this				_X1	_X2	_X1	_X2	_X1	_X2
	study)									
Number of amino acids	386	386	386	382	386	324	382	382	382	382
Molecular weight (kDa)	43.36	43.26	43.26	43.26	43.26	36.32	42.85	42.85	42.81	43.04
Isoelectric point (PI)	6.24	6.34	6.39	6.34	6.39	9.10	6.24	6.24	6.24	5.98
Negatively charged	51	50	50	50	50	36	50	50	50	48
residues (Asp + Glu)										
Positively charged	47	47	47	47	47	47	46	46	46	42
residues (Arg + Lys)										
Hydrophobic amino	97	97	97	97	97	76	97	97	97	104
acids (A, I, L, F, W, V)										
Polar amino acids	112	112	111	112	111	92	109	109	109	112
(N, C, Q, S, T, Y)										
Instability index (II)	78.70	80.27	78.29	80.27	78.29	78.85	77.09	77.09	80.22	77.87
Grand average of	-0.730	-0.727	-0.726	-0.727	-0.726	-0.774	-0.711	-0.711	-0.712	-0.616
hydropathicity (GRAVY)										
Aliphatic index (AI)	63.42	62.93	62.93	62.93	62.93	57.81	62.04	62.04	62.04	67.15

**Table 3:** The functional modification sites harbored in buffalo TP53.

Name of modification site	Serial no.	Location and amino composition
Casein kinase II phosphorylation site	PS00006	4–7: SqaE, 18–21: TfsD, 51–54: TwlD, 249–252:
		TleD, 277–280: TeeE, 288–291: ScpE
Protein kinase C phosphorylation site	PS00005	91–93: SqK, 110–112: TaK, 204–206: TfR, 296–298:
		StK, 297–299: TkR, 364-366: SkK
N-myristoylation site	PS00008	109–114: GtakSV, 192–197: GnlrAE, 255–260: GNllGR
N-glycosylation site	PS00001	232–235: NSSC, 304–307: NTSS
Tyrosine kinase phosphorylation site 2	PS00007	313-320: KkrlDeeY
cAMP- and cGMP-dependent protein	PS00004	366-369: KRpS
kinase phosphorylation site		

### 3.4 Motifs, conserved domains, and evolutionary relationship

**Figure 4:**
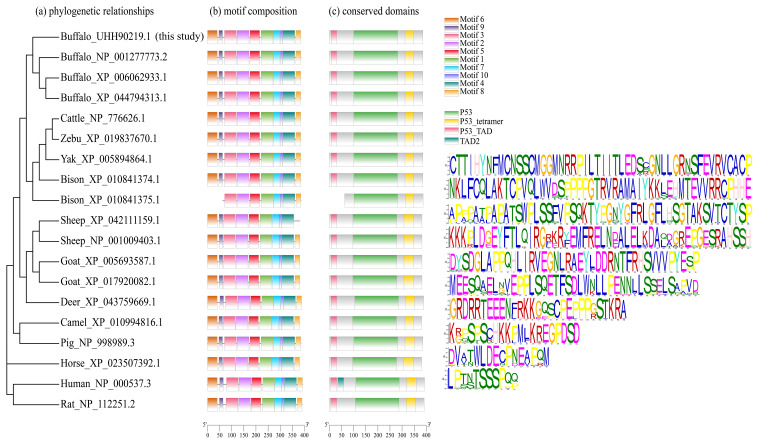
Phylogenetic relationship, motif composition, and conserved domains of TP53 in buffalo and other species.

The motif composition and conserved domains of multiple mammalian TP53 proteins were analyzed, and a total of 10 motifs and 4 conserved domains were identified (Fig. 4). The DBD (AA101-282) of buffalo TP53 is composed of motifs 1, 2, 4, 5, and 7. The TD (AA312-348) is composed of motif 3, and the TAD (AA7-30) is composed of motif 6. The protein encoded by the transcript variant X2 in bison lacks motif 6, motif 9, and domain TAD. The TP53 protein lacking the domain TAD can inhibit the transcriptional activation of the normal TP53 protein, mediate the export of the intact TP53 from the nucleus to the cytoplasm, and undergo monoubiquitination in an MDM2-independent manner (Ghosh et al., 2004). The transcription variant X2 of sheep TP53 lacks motif 8 but does not affect the conserved domain composition of the protein. In human, the TAD is composed of TAD1 and TAD2 (Raj and Attardi, 2017). A phylogenetic tree constructed based on TP53 amino acid sequences with rat and human as the outgroup is shown in Fig. 4. The results show that buffalo are clustered with the animals of the *Bos* genus, suggesting that buffalo TP53 has a similar function to that of such animals.

### 3.5 Secondary and three-dimensional structure

The secondary structure composition of buffalo TP53 is highly similar to that of other species in Bovidae, except that of the TP53 transcript variant X2 in bison and sheep (Fig. S3 and Table S3). The predicted three-dimensional structure of buffalo TP53 is highly similar to that of other species in Bovidae, except that of the bison TP53 transcript variant X2 (Fig. S4).

### 3.6 Proteins interacting with buffalo TP53

**Figure 5:**
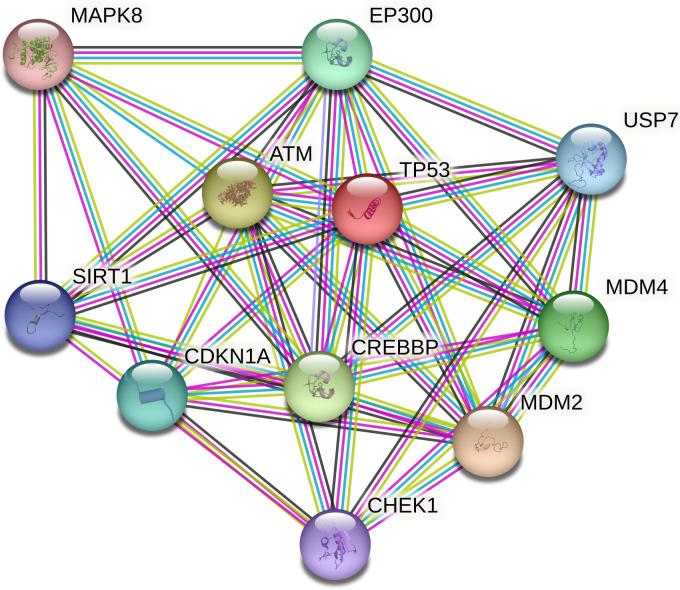
Protein–protein interaction network of buffalo TP53. Several putative interactors were predicted for TP53 protein. The colors of the lines represent the different modes by which the interaction was predicted. Light-green color is for text mining, red line color is for gene fusions, purple color is for experimentally determined, and cyan color indicates attainment from curated databases.

Ten proteins were predicted to interact with buffalo TP53 (Fig. 5). There are three kinds of proteins in the network: one is related to lipid synthesis, which includes MDM2 (murine double minute 2), CDKN1A, and EP300 (E1A-binding protein P300); the other is related to protein synthesis, which includes ATM (ataxia–telangiectasia mutated) and SIRT1 (silent information regulator 1); and another is associated with apoptosis, which includes MAPK8 (mitogen-activated protein kinase 8), MDM4 (murine double minute 4), USP7 (ubiquitin-specific peptidase 7), and CHEK1 (checkpoint kinase 1).

### 3.7 Subcellular localization, biological processes, and molecular function

The buffalo and cattle TP53 proteins were predicted to be localized in the nucleus (100 %) and involved in the biological processes of apoptosis (GO: 0006915), biological rhythms (GO: 0048511), cell cycle (GO: 0007049), transcription, transcription regulation, and necrosis. They are mainly involved in the molecular functions of DNA-binding transcription factor activity (GO: 0003700), DNA binding (GO: 0003677), transcription cis-regulatory region binding (GO: 0000976), and metal ion binding (GO: 0046872). In addition, they participate in the MAPK signal pathway, TP53 signal pathway, PI3K–AKT signal pathway, cell cycle, apoptosis, and cell senescence.

### 3.8 Tissue differential expression

**Figure 6:**
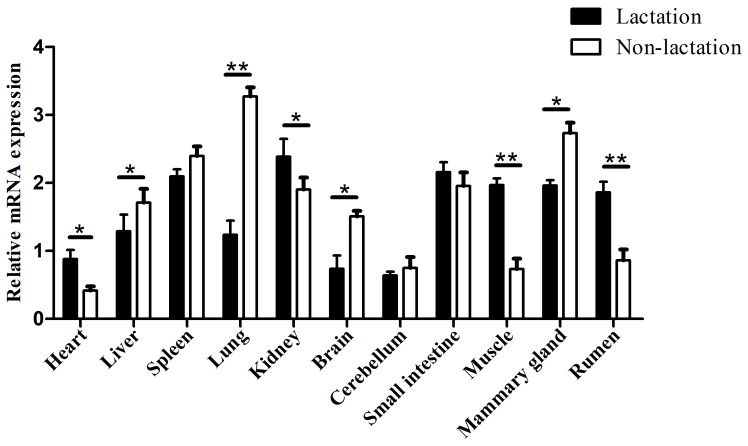
Tissue differential expression of *TP53* in 11 buffalo tissues during lactating and non-lactating stages. The values are presented as means ± SEM; ${}^{*}$ – p<0.05 ; ${}^{**}$ – p<0.01 .

To ensure that the results of the qPCR detection were the target gene, all amplification products were verified by sequencing and BLAST alignments (Table S4). Among the 11 buffalo tissues of lactation and non-lactation detected, *TP53* was found to express in all tissues (Fig. 6). In the non-lactation period, its expression levels were relatively high in the lung, mammary gland, and spleen, while in the lactation period, its expression was relatively high in the kidney, small intestine, spleen, muscle, mammary gland, and rumen. It is worth noting that the expression of *TP53* in the heart, kidney, brain, muscle, and rumen during lactation was significantly higher than that during non-lactation ( p<0.05 ), while its expression in the liver, lung, and mammary gland was just the opposite ( p<0.05 ).

### 3.9 Knockdown of buffalo *TP53* promotes the synthesis of milk protein and milk fat in BuMECs

Here, the interference efficiency of two specific siRNAs targeting the *TP53* CDS was evaluated in BuMECs. Compared with negative control (siRNA-NC)-treated cells, the mRNA abundance of this gene was significantly reduced in cells transfected with specific siRNAs, with an 87 % reduction for siRNA1-TP53 and a 69 % reduction for siRNA2-TP53 ( p<0.05 ; Fig. S5). Therefore, we transfected siRNA1-TP53 into BuMECs for further functional experiments due to its higher interference efficiency.

**Figure 7:**
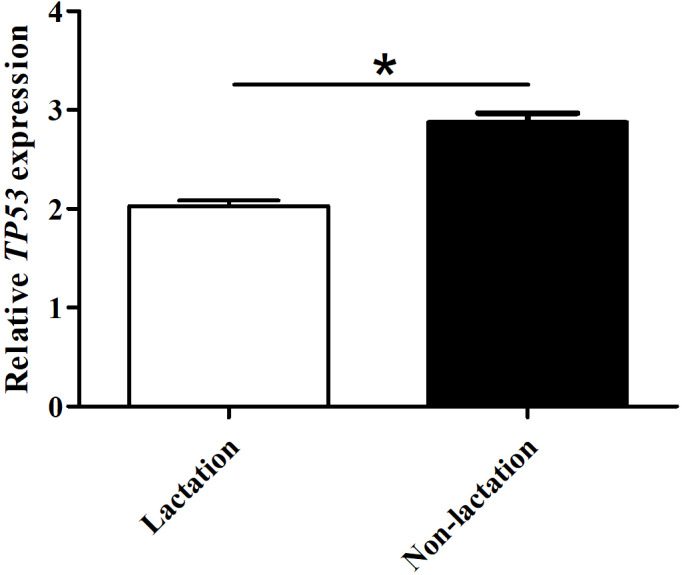
The mRNA abundance of the *TP53* gene in lactating and non-lactating BuMECs. The values are presented as means ± SEM; ${}^{*}$ – p<0.05 ; ${}^{**}$ – p<0.01 .

The mRNA abundance of *TP53* in lactating and non-lactating BuMECs was examined by qPCR. The results showed that the mRNA abundance of *TP53* in BuMECs was significantly lower during lactation than during non-lactation ( p<0.0 5; Fig. 7). In order to determine whether *TP53 *expression is involved in the synthesis of buffalo milk protein and milk fat, we performed knockdown experiments on TP53 by transfecting siRNA1-TP53 into BuMECs.

**Figure 8:**
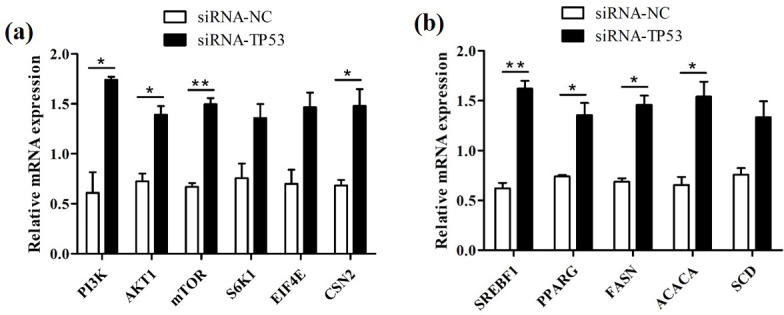
The effects of *TP53* knockdown on genes related to milk protein and milk fat synthesis in BuMECs. **(a)** The effect of *TP53 *knockdown on the expression of genes related to milk protein synthesis. **(b)** The effect of *TP53* knockdown on the expression of genes related to milk fat synthesis. The values are presented as means ± SEM; ${}^{*}$ – p<0.05 ; ${}^{**}$ – p<0.01 .

We successfully knocked down the *TP53* gene in BuMECs and further detected changes in the expression levels of genes related to milk protein and milk fat synthesis in BuMECs under *TP53 *gene knockdown. The knockdown of *TP53* led to changes in the expression of the *PI3K*, *AKT1*, *mTOR*, *S6K1*, *EIF4E*, and *CSN2* genes related to milk protein synthesis, as well as in the *SREBF1*, *PPARG*, *ACACA*, *FASN*, and *SCD* genes related to milk fat synthesis. In comparison to the group of negative control (siRNA-NC), the expressions of the *PI3K*, *AKT1*, *mTOR*, *S6K1*, *EIF4E*, and *CSN2* genes were increased 1.85-fold ( p<0.05 ), 0.93-fold ( p<0.05 ), 1.23-fold ( p<0.0 1), 0.80-fold, 1.10-fold, and 1.17-fold ( p<0.05 ), respectively, in the group treated with siRNA1-TP53 (Fig. 8a). In addition, the *SREBF1*, *PPARG*, *ACACA*, *FASN*, and *SCD* genes increased 1.61-fold ( p<0.01 ), 0.83-fold ( p<0.05 ), 1.12-fold ( p<0.05 ), 1.34-fold ( p<0.05 ), and 0.76-fold, respectively (Fig. 8b).

### 3.10 Population variation

**Figure 9:**
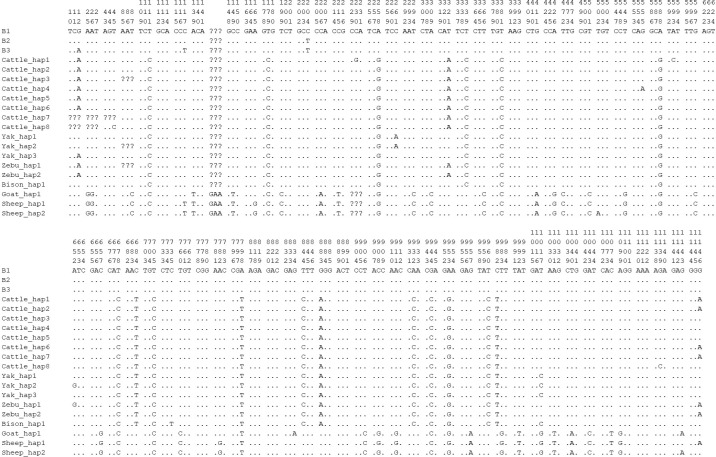
Differential sites for *TP53* nucleotide sequences between buffalo and other species of Bovidae. Numbers indicate the position of the coding region. Point ( ⋅ ) indicates consistency with the sequence B1. Nucleotide substitutions are represented by different letters. Missing information is marked with a question mark (?). A horizontal line (–) indicates a deletion in the sequence.

In this study, one synonymous substitution was found in the *TP53* CDS of river buffalo, that is, c.204C > T, which had three genotypes of CC, CT, and TT, with a high frequency of C allele, while the CC homozygote was found at c.204 in swamp buffalo. Codon analysis of the *TP53* gene showed little difference in codon preference between the sequence (B1, this study) of c.204C and the sequences (XM_006062871 and NM_001290844 from the NCBI database, named B2 and B3, respectively) of c.204T (Table S5). The sequence B3 was found to have two nucleotide differences compared to the sequences B1 and B2 at c.12 and c.117 (Fig. 9).

### 3.11 Nucleotide sequence differences in Bovidae species

The sequences of buffalo *TP53* in this study were aligned with the *TP53* sequences of Bovidae species downloaded from the NCBI database (Fig. 9 and Table S6; only one sequence was taken if they were identical). There are 17 nucleotide difference sites between buffalo and other species of Bovidae, which are c.111, c.179, c.204, c.258, c.339, c.588, c.678, c.684, c.705, c.798, c.844, c.883, c.932, c.944, c.953, c.960, and c.982. Among them, six nucleotide sites of c.179, c.844, c.883, c.944, c.953, and c.982 lead to the amino acid differences of TP53 protein sequences (Fig. S6). It is noteworthy that there is an InDel (insertion–deletion) codon in the *TP53* gene between buffalo, the *Bos* genus, and the *Ovis *genus.

## 4 Discussion

In this study, the complete coding sequence of the buffalo *TP53* gene was isolated and identified. An mRNA transcript of buffalo *TP53* was obtained, and its CDS encodes a polypeptide consisting of 386 amino acid residues, which is consistent with a previous report (Singh et al., 2015). The TP53 protein of buffalo is a stable hydrophilic protein without a signal peptide and transmembrane domain, which plays a transcriptional regulatory role in the nucleus as a transcription factor. The alignment of sequences revealed that, except for the molecular characteristics of the TP53 transcript variant X2 in bison and sheep, the structure of the transcriptional region of *TP53*, the basic physicochemical characteristics, secondary structure composition, tertiary structure, motif composition, and conserved domains of the TP53 protein in species of Bovidae are highly consistent, although there are some interspecies differences. Phylogenetic analysis showed that buffalo and the livestock in the *Bos* genus were clustered together, which indicates that the function of the buffalo TP53 is more similar to those of animals in the *Bos* genus. Previous study has found that mouse TP53 contains three domains of TAD, DBD, and TD (Lane, 1992), which are shared by the members of the TP53 family (Kaelin, 1999). The TAD domain is required for TP53 to achieve transcriptional activation activity and can interact with a variety of proteins, including the transcription factors TFIID and TFIIH, as well as several TATA box-binding protein-associated factors (TAFs) (Kamada et al., 2016). The TD domain can regulate the TP53 oligomeric state and DNA-binding activity, and the DBD enables TP53 to bind to relevant DNA sequences to play a functional role (Kamada et al., 2016). In this study, we found that buffalo TP53 also possesses the three conserved domains that are consistent with those found in mice. Comparative analysis suggests that the function of TP53 is conservative. It is functionally consistent in mammals, and that of buffalo is particularly consistent with that of the species of the *Bos* genus.

The post-translational modification of the TP53 protein is an important mechanism by which TP53 responds to a variety of signal stimuli, including phosphorylation, ubiquitination, acetylation, and methylation (Kruse and Gu, 2008). Phosphorylation modification of TP53 is a key step to stabilize TP53 and enhance TP53 transcriptional activity (Nguyen et al., 2014). Activation of PKC by genotoxic stress induces phosphorylation of TP53 on Ser ${}^{46}$ , resulting in the commitment of cell fate to apoptosis (Yoshida et al., 2006). The casein kinase 2 (CK2) can phosphorylate the C-terminal Ser ${}^{392}$ of TP53, resulting in an increase in the specific binding of TP53 to DNA (Leblanc and May, 2002). In this study, we found that buffalo TP53 also has phosphorylation sites of PKC and CK2, but whether these sites play a functional role in the post-transcriptional regulation of buffalo TP53 remains to be further investigated.

Previous study in mice has found that *TP53* is highly expressed only in the brain, liver, lung, intestine, kidney, thymus, and salivary glands during the differentiation stage of various organ tissues but not in other organs and tissues (Schmid et al., 1991). In this study, the differential expression analysis of *TP53* displayed that *TP53* was expressed in all 11 buffalo tissues during lactation and non-lactation, which indicates that this gene plays a functional role in multiple tissues of buffalo. The expression of buffalo *TP53* in the heart, kidney, brain, muscle, and rumen during lactation was significantly higher than that during non-lactation, while the expression of *TP53* in the liver, lung, and mammary gland was the opposite, which might be related to the different physiological states of buffalo. It is worth noting that the mRNA abundance of *TP53* in BuMECs was also lower during lactation than during non-lactation. It has been shown that the knockdown of *TP53* leads to delayed degeneration of mammary epithelial cells in mice (Jerry et al., 1998). Therefore, it can be inferred that buffalo TP53 is related to lactation.

In this study, buffalo TP53 was predicted to interact with ATM and SIRT1. Study has reported that cytoplasmic ATM can regulate protein synthesis in the insulin signaling pathway by phosphorylating 4E-BP1 (Yang and Kastan, 2000). SIRT1 negatively regulates the mammalian target of rapamycin (mTOR) signaling through the tuberous sclerosis complexes 1 and 2 (TSC1 and TSC2) (Ghosh et al., 2010). It is therefore hypothesized that buffalo TP53 is involved in protein synthesis. In mammals, milk protein synthesis is regulated by the expression of genes downstream of the mTOR pathway associated with milk protein synthesis, such as *S6K1* and *eIF4E *(Rius et al., 2010; Wang et al., 2006). The *eIF4E* and *S6K1* genes play an essential role in the translation of ribosomal mRNA sequences, and mTOR ultimately participates in protein synthesis by affecting their expression levels (Hayashi et al., 2009; Saxton and Sabatini, 2017). β -casein (CSN2) is the second most abundant protein in cows' milk and can be used as an indicator of protein synthesis ability in bovine mammary epithelial cells (BMECs) (Chatchatee et al., 2001). However, TP53 can induce the expression of genes that negatively regulate the insulin-like growth factor 1 (IGF1)–protein kinase B (AKT) pathway and mTOR pathway, which include phosphatase and tension homolog (*PTEN*), *TSC*, and AMP-activated protein kinase (*AMPK*) genes (Zhang et al., 2010). In this study, the mRNA abundance of phosphoinositide 3-kinase (*PI3K*), *AKT1*, *mTOR*, *S6K1*, *EIF4E*, and *CSN2* genes associated with milk protein synthesis were increased after interfering with *TP53* in BuMECs. It has been reported that TP53 inhibits the synthesis of milk protein through the mTOR pathway in the lactating mammary gland of dairy cows (Bionaz and Loor, 2011). Therefore, the results can indicate that buffalo *TP53* inhibits milk protein synthesis.

Proteins predicted to interact with buffalo TP53 include MDM2, CDKN1A, EP300, SIRT1, and ATM in this study. MDM2 is an E3 ubiquitin ligase with carcinogenic effect, which can promote adipogenesis by enhancing CREB-mediated trans-activation and limiting TP53 activity (Hallenborg et al., 2012). TP53 was found to induce the expression of CDKN1A, which promotes lipid accumulation in mouse liver and white adipose tissue (Takasaki et al., 2012) and leads to an increase in the apoptosis threshold in the cells of human lung cancer (Marijn et al., 2019). EP300 (P300) is an acetyltransferase that transcriptionality upregulates the expression of fatty acid synthase (FASN) and promotes the accumulation of lipids (Gang et al., 2016). SIRT1 regulates lipid homeostasis in mouse liver by positively regulating the peroxisome proliferator-activated receptor α (PPAR α ) (Purushotham et al., 2009). The *ATM* gene encodes a serine–threonine protein kinase of the phosphatidylinositol-3 kinase-related protein kinase (PIKK) superfamily (Khalil et al., 2012), which has been reported to be highly expressed in well-differentiated adipocytes in pigs, suggesting that it may participate in adipocyte differentiation (Wang and Proud, 2006). It is therefore hypothesized that buffalo TP53 is involved in the process of fat metabolism. In mammary tissues, sterol regulatory element-binding proteins (SREBPs) and PPAR γ are important transcriptional regulators in the fatty acid synthesis gene network, directly regulating genes such as *FASN*, acetyl-CoA carboxylase alpha (*ACACA*), and stearoyl-CoA desaturase (*SCD*) that affect milk fat transport and synthesis (Carreño et al., 2016; Liu et al., 2016). FASN, SCD, and ACACA are central enzymes for milk fat synthesis. In this study, the mRNA abundance of the *SREBF1*, *PPARG*, *ACACA*, *FASN*, and *SCD* genes involved in milk lipid synthesis were elevated after knockdown of *TP53* in BuMECs. Previous study showed that TP53 inhibits the expression of *SREBF1* and *FASN* genes in the adipocytes of mice, thereby inhibiting the synthesis of fat (Yahagi et al., 2003). Therefore, the results showed that TP53 inhibited the synthesis of milk fat in buffalo. This study predicts that buffalo TP53 participates in the PI3K–AKT pathway. This pathway is the upstream of the mTOR pathway and plays a crucial role in protein and lipid synthesis (Appuhamy, 2010; Smith et al., 2008). Studies have also found that the mTOR pathway can affect the expression of SREBPs and PPAR γ , thereby regulating fatty acid synthesis (Li et al., 2017; Huang et al., 2020; Caron et al., 2015). These results indicate that *TP53* negatively regulates the synthesis of milk protein and milk fat in BuMECs through the PI3K–AKT–mTOR pathway.

In mice, a study showed that the substitution of arginine for leucine at the position 172 of TP53 in the mammary gland can lead to lactation failure (Li et al., 1994). In this study, an SNP (c.204C > T) was found in the coding region of *TP53* in river buffalo, where the nucleotide substitution is synonymous and does not affect the preference of codon usage; thus, it is presumed to have no effect on the function of buffalo TP53. In addition, there are 17 nucleotide difference sites in CDS between buffalo and other species in Bovidae, 6 of which lead to amino acid differences. These sites can be used as molecular markers to distinguish buffalo from other species of Bovidae.

## 5 Conclusions

The molecular characteristics and function of the buffalo *TP53* gene and its encoded protein are highly similar to those of other species in Bovidae, indicating that it is functionally conserved. It plays a functional role as a transcription factor in the nucleus and is involved in metabolic regulation related to the PI3K–AKT pathway. *TP53 *plays an important functional role in multiple tissues of buffalo and negatively regulates milk protein and milk fat synthesis in the tissue of the mammary gland through the PI3K–AKT–mTOR pathway. The synonymous substitution (c.204C > T) found here in river buffalo has limited potential value in buffalo-breeding selection. The results of this study provide new insights into the functional role of *TP53* in the lactation of buffalo.

## Supplement

10.5194/aab-67-217-2024-supplement The supplement related to this article is available online at: https://doi.org/10.5194/aab-67-217-2024-supplement.

## Data Availability

All relevant data are available within this paper and its Supplement.
