# Supplementary material for: Molecular characterization, function, tissue differential expression, and single-nucleotide polymorphism of buffalo TP53 gene
Source: Arch Anim Breed. 2024 May 21;67(2):217–30. doi: 10.5194/aab-67-217-2024 (PMC13151978; doi:10.5194/aab-67-217-2024)
Supplement: The supplement related to this article is available online at: https://doi.org/10.5194/aab-67-217-2024-supplement. [file aab-67-217-2024-supplement.pdf]

Supplement of Arch. Anim. Breed., 67, 217–230, 2024
https://doi.org/10.5194/aab-67-217-2024-supplement
© Author(s) 2024. CC BY 4.0 License.

Open Access
Archives Animal Breeding

Supplement of

Molecular characterization, function, tissue differential expression, and single-nucleotide polymorphism of buffalo *TP53* gene

Lige Huang et al.

Correspondence to: Yongwang Miao (yongwangmiao1@126.com)

The copyright of individual parts of the supplement might differ from the article licence.

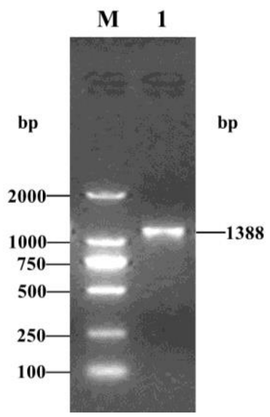

Figure S1 PCR product of buffalo *TP53*. M, DL2000 DNA Marker; 1, the sample from the mammary gland

Percent Identity (upper triangle) / **Divergence** (lower triangle)

	1	2	3	4	5	6	7	8	9	10	11		
**1**		99.7	98.3	98.4	98.3	98.4	98.3	96.2	96.2	96.0	93.0	**1**	Buffalo_OL456217(this study)
**2**	0.3		98.2	98.1	98.2	98.1	98.2	96.0	96.0	95.9	92.9	**2**	Buffalo_NM_001290844.2_X1
**3**	1.7	1.8		99.6	100.0	99.6	99.6	96.2	96.2	96.0	93.0	**3**	Cattle_NM_174201.2
**4**	1.7	1.9	0.4		99.6	99.8	99.8	96.4	96.4	96.2	93.2	**4**	Yak_XM_005894802.2
**5**	1.7	1.8	0.0	0.4		99.6	99.6	96.2	96.2	96.0	93.0	**5**	Zebu_XM_019982111.1
**6**	1.7	1.9	0.4	0.2	0.4		100.0	96.4	96.4	96.2	93.2	**6**	Bison_XM_010843072.1_X1
**7**	1.8	1.9	0.4	0.2	0.4	0.0		96.2	96.2	96.1	92.6	**7**	Bison_XM_010843073.1_X2
**8**	3.9	4.1	3.9	3.7	3.9	3.7	3.9		100.0	99.6	96.4	**8**	Goat_XM_005693530.3_X1
**9**	3.9	4.1	3.9	3.7	3.9	3.7	3.9	0.0		99.6	96.4	**9**	Goat_XM_018064593.1_X2
**10**	4.1	4.2	4.1	4.0	4.1	4.0	4.0	0.4	0.4		96.7	**10**	Sheep_NM_001009403.1_X1
**11**	7.3	7.4	7.3	7.1	7.3	7.1	7.8	3.7	3.7	3.4		**11**	Sheep_XM_042255225.1_X2
	**1**	**2**	**3**	**4**	**5**	**6**	**7**	**8**	**9**	**10**	**11**		

Figure S2 Consistency between the CDS sequence obtained in this study and the CDS sequences of *TP53* in NCBI database

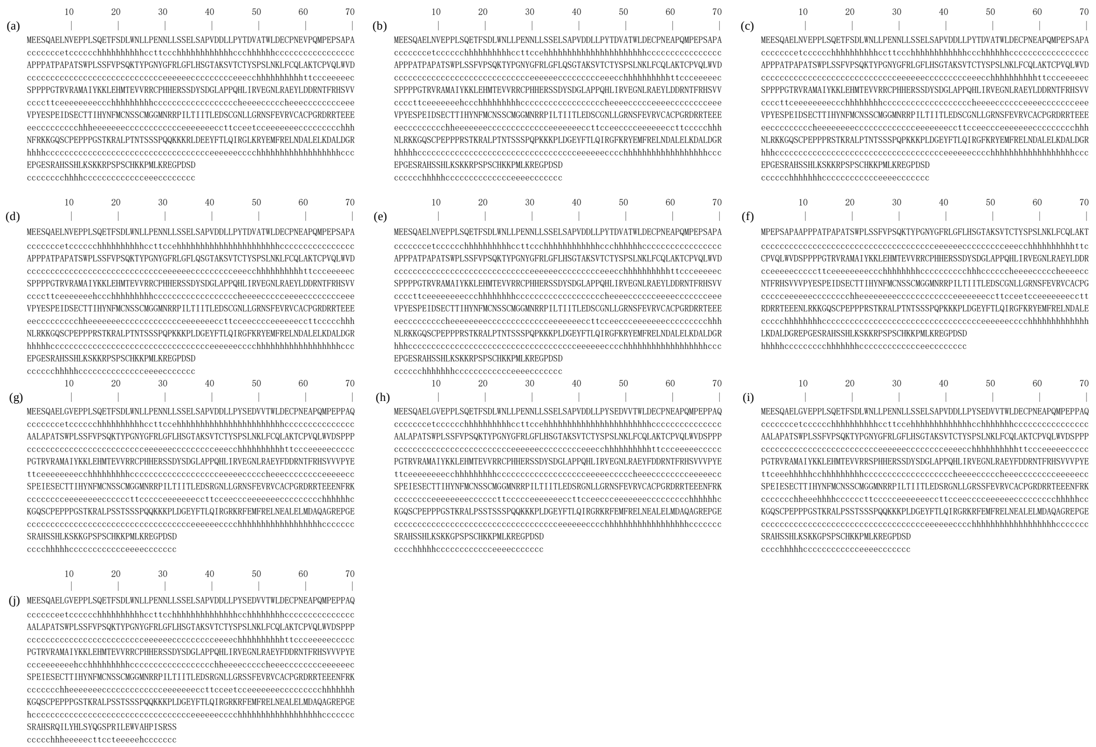

Figure S3 The secondary structure of TP53 for buffalo and other species of Bovidae. The composition of secondary structure of TP53 is represented by a single letter below its amino acid sequence: h, alpha helix; e, extended strand; t, beta turn; c, random coil. (a), Buffalo (UHH90219.1); (b), Cattle (NP_776626.1); (c), Yak (XP_005894864.1); (d), Zebu (XP_019837670.1); (e), Bison (XP_010841374.1); (f), Bison (XP_010841375.1); (g), Goat (XP_017920082.1); (h), Goat (XP_005693587.1); (i), Sheep (NP_001009403.1); (j), Sheep (XP_042111159.1).

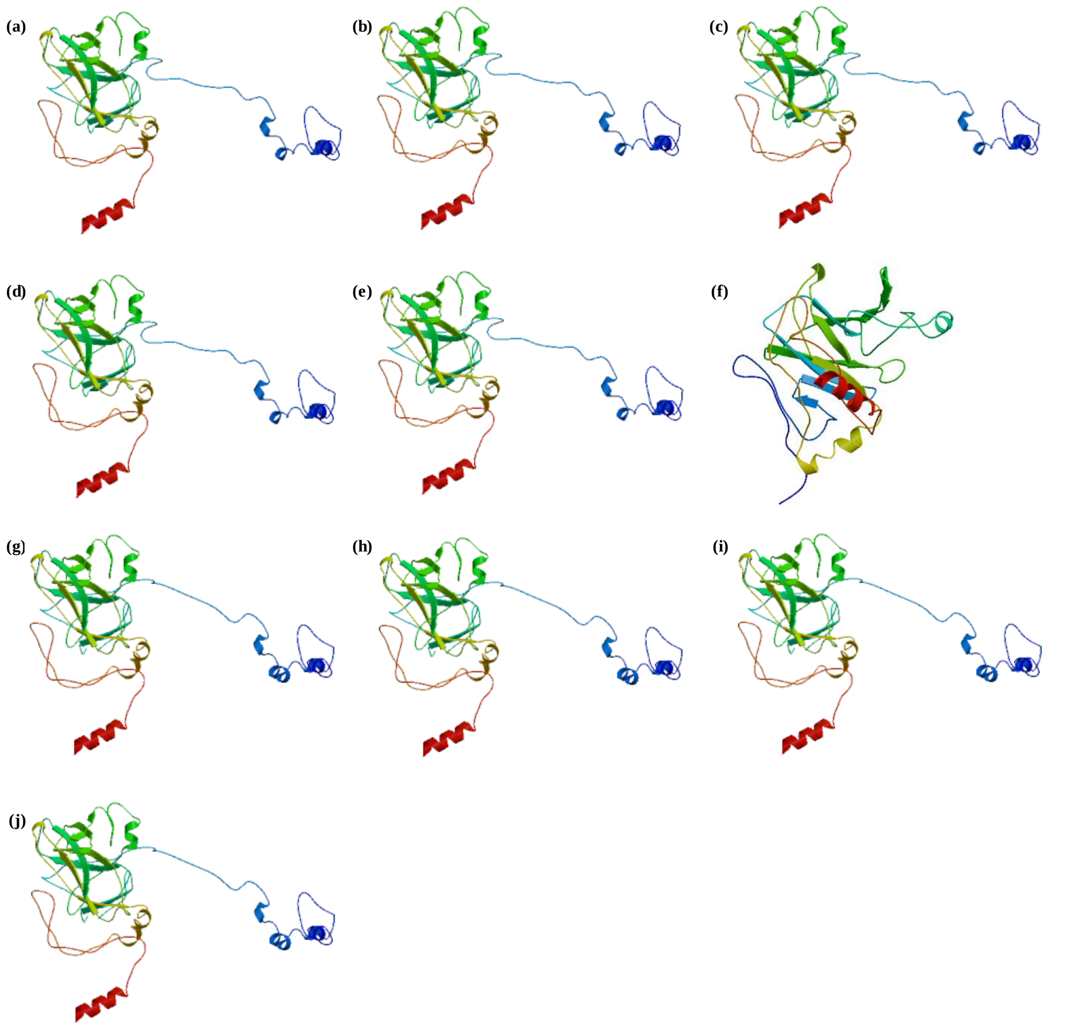

Figure S4 The three-dimensional structures of TP53 for buffalo and other species of Bovidae. The coverage rate of TP53 sequence in buffalo (UHH90219.1), cattle (NP_776626.1), yak (XP_005894864.1), zebu (XP_019837670.1), bison (XP_010841374.1), goat (XP_017920082.1), goat (XP_005693587.1), sheep (NP_001009403.1) with template 6xre.1.M (human) sequence was 100 %, and the sequence consistency was 80.26%, 81.30%, 81.56%, 81.30%, 81.56%, 81.10%, 81.10% and 80.58%, respectively. While, the coverage rate of TP53 sequence in bison (XP_010841375.1) with template 6xre.1.M sequence was 98%, and the sequence consistency was 86.44%. The coverage rate of TP53 sequence in sheep (XP_042111159.1) with the sequence of template 6xre.1.M was 93%, and the sequence consistency was 81.07%. (a), Buffalo (UHH90219.1); (b), Cattle (NP_776626.1); (c), Yak (XP_005894864.1); (d), Zebu (XP_019837670.1); (e), Bison (XP_010841374.1); (f), Bison (XP_010841375.1); (g), Goat (XP_017920082.1); (h), Goat (XP_005693587.1); (i), Sheep (NP_001009403.1); (j), Sheep (XP_042111159.1).

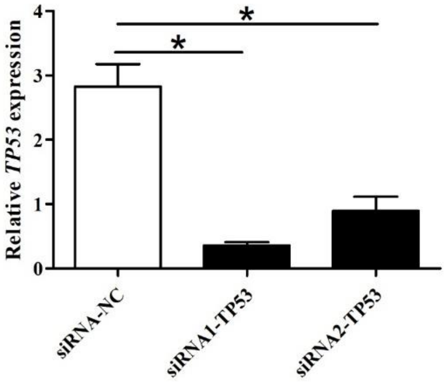

Figure S5 Evaluation of two pairs of siRNAs for *TP53* interference efficiency in BuMECs. The values are presented as means ± SEM; *, $p < 0.05$; **, $p < 0.01$.

```
                        11111222222222222333333333333333
                    34 5666770689901156677899000111233444678
                   987 0079277838988940134257234158819478740
B1            NAT-AVSPPPHCPYLSIDCRNRDFGTPTNQRELYDKLDRKR
B2            ...-.....................................
B3            ...-.....................................
Cattle_hap1   ...-.A...RQ..S.........LR....PPGF........
Cattle_hap2   ...-.A....Q............LR....PPGF........
Cattle_hap3   ...-.A....Q............LR....PPGF........
Cattle_hap4   ...-.A....Q............LR....PPGF........
Cattle_hap5   ...-.A....Q............LR....PPGF........
Cattle_hap6   ...-.A....Q............LR....PPGF........
Cattle_hap7   ?..-.A....Q............LR....PPGF........
Cattle_hap8   ?..-.A....Q............LR....PPGF.......T
Yak_hap1      ...-.A.................LR....PPGF........
Yak_hap2      ...-.A..........V......LR....PPGF........
Yak_hap3      ...-.A.................LR....PPGF........
Zebu_hap1     ...-.A....Q............LR....PPGF........
Zebu_hap2     ...-.A....Q.....V......LR....PPGF........
Bison_hap1    ...-.A.................LR....PPGF........
Goat_hap1     G.SEVAPQL?....F..ER........SS.PGRFEMQAG..
Sheep_hap1    G.SEVAPQL?....F..ER.S......SS.PGRFEMQAG..
Sheep_hap2    G.SEVAPQL?.S..F..ER.S......SS.PGRFEMQAG..
```

Figure S6 Differential sites for amino acid sequences of TP53 between buffalo and other species of Bovidae. Numbers indicate the position of the coding region. Point (·) indicates consistency with the B1. Nucleotide substitutions are represented by different letters. Missing information is marked with a question mark (?). A horizontal line (-) indicates a deletion in the sequence.

Table S1 The information of primers used in this study

Gene	Serial number of template	Primer sequence (5’→3’)*	Product length (bp)	Annealing temperature (℃)	Extension time (s)	Purpose
TP53	XM_006062871	F: CGGGGTCATCTAGCGTCC<br>R: CACCAAGCAGAGGTCTAAGCA	1388	63.9	84	Gene cloning
TP53	OL456217	F: ATGGAAGAATCGCAGGCAGAACT<br>R: ATTTGGGGCACTTCATTCGGACA	188	60.2	12	Expression level detection
siRNA1-TP53	OL456217	F: GGAAUUUACGCGCAGAGUATT<br>R: UACUCUGCGCGUAAAUUCCTT	/	/	/	Gene interference
siRNA2-TP53	OL456217	F: GCUCCCGUACACAGAUGUUTT<br>R: AACAUCUGUGUACGGGAGCTT	/	/	/	Gene interference
siRNA-NC	/	F: UUCUCCGAACGUGUCACGUTT<br>R: ACGUGACACGUUCGGAGAATT	/	/	/	Negative control
PI3K	XM_006069974	F: CAGTCACCTCTCAACCCA<br>R: GCCGTAAATCATCACCAT	160	60	30	Expression level detection
AKT1	NM_001290841	F: CCAAGTCCCTGCTCTCGG<br>R: TTCGCTGTCCACCCCCTC	266	60	30	Expression level detection
mTOR	XM_025285881	F: ATGCTGTCCCTGGTCCTTATG<br>R: GGGTCAGAGAGTGGCCTTCAA	178	60	30	Expression level detection
EIF4E	NM_001319800	F: GATGGGCACTCTGGTTTT<br>R: CCTCCTCGTTTGTTTTTC	208	60	30	Expression level detection
S6K1	XM_006065441	F: TGCTGCTTCTCGTCTTGG<br>R: GGTTGGCACTTTCACTGA	224	60	30	Expression level detection
CSN2	XM_006071124	F: GGCTCCTAAGCACAAAGA<br>R: AGGGGGAAACATGACAGT	172	60	30	Expression level detection
SREBF1	XM_025280442	F: GCACCGAGGCCAAGTTGAATAA<br>R: CAGGTCCTTCAGCGATTTGCTT	146	57	30	Expression level detection
PPARG	NM_001290893	F: GCTCCAAGAGTACCAAAGTG<br>R: GTCCTCCTGAAGAAACCCTT	204	53.7	30	Expression level detection
FASN	XM_006061793	F: AGGCCAGCTCCGAAGGCAACA<br>R: TACCACGTCGGCCACTTGTGTC	209	64.3	30	Expression level detection
ACACA	XM_025281124	F: CCTCTTCAGACAGGTTCAAGC<br>R: TTCACCGCACACTGTTCCA	234	55	30	Expression level detection
SCD	NM_001290915	F: CGTGCCGTGGTATCTGTGG<br>R: AAAGGTGTGGTGGTAGTTGTGG	217	56.2	30	Expression level detection
ACTB	NM_001290932	F: TCTTGGTCACCTCGTCGTC<br>R: GGCGCGATGATCTTGAT	145	59.5	9	Internal reference
GAPDH	XM_006065800	F: ATGGAGAAGGCTGGGGCTCA<br>R: GCAGGAGGCATTGCTGACAA	144	60	9	Internal reference
RPS23	XM_006059350	F: ACCGACGAGACCAGAAGT<br>R: CTCCAGGAATGTCACCAA	306	60	19	Internal reference
Exon 1	NC_059159	F: GTTACGGTTTTCGCTTGT<br>R: GAGTTAGTGGTGGTGGGG	671	61.8	41	SNP detection
Exon 2-4	NC_059159	F: GGTGTGCTGTGGGCGGAG<br>R: AGGCAAAGGCTGAAGTGG	795	65.5	48	SNP detection
Exon 5-7	NC_059159	F: GTTTTCATCCTTCCCGTC<br>R: AATAACCCATCTGCTCTG	753	59.2	46	SNP detection
Exon 8-10	NC_059159	F: ACCACATTCTCCCACACT<br>R: AGACCTGGATCACTCACC	1099	61.4	66	SNP detection
Exon 11	NC_059159	F: CCTCCCTTTTCTCCTCTC<br>R: GCTTCCCAAACTCACTCC	1027	58.6	62	SNP detection

*, Primer direction (F: forward; R: reverse).

Table S2 Sequence information of *TP53* gene

Species	Accession number of nucleotide sequences	Accession number of protein sequences	CDS length (bp)	CDS status
Buffalo (this study)	OL456217	UHH90219.1	1158	Complete
Buffalo_X1	NM_001290844.2	NP_001277773.2	1158	Complete
Buffalo_X2	XM_006062871.2	XP_006062933.1	1158	Complete
Buffalo_X3	XM_044938378.1	XP_044794313.1	1158	Complete
Cattle	NM_174201.2	NP_776626.1	1158	Complete
Yak	XM_005894802.2	XP_005894864.1	1158	Complete
Zebu	XM_019982111.1	XP_019837670.1	1158	Complete
Bison_X1	XM_010843072.1	XP_010841374.1	1158	Complete
Bison_X2	XM_010843073.1	XP_010841375.1	972	Complete
Goat_X1	XM_005693530.3	XP_017920082.1	1146	Complete
Goat_X2	XM_018064593.1	XP_005693587.1	1146	Complete
Sheep_X1	NM_001009403.1	NP_001009403.1	1146	Complete
Sheep_X2	XM_042255225.1	XP_042111159.1	1146	Complete
Pig	NM_213824.3	NP_998989.3	1158	Complete
Deer	XM_043903734.1	XP_043759669.1	1167	Complete
Horse	XM_023651624.1	XP_023507392.1	1142	Complete
Camel	XM_010996514.2	XP_010994816.1	1143	Complete
Rat	NM_030989.3	NP_112251.2	1173	Complete
Human	NM_000546.6	NP_000537.3	1179	Complete

Table S3 Secondary structure composition of TP53 in some domestic animals of Bovidae

Structer	Buffalo (this study)	Cattle	Yak	Zebu	Bison _X1	Bison _X2	Goat _X1	Goat _X2	Sheep _X1	Sheep _X2
Random coil (%)	59.07	57.25	59.07	57.25	59.07	58.33	57.07	57.07	57.85	54.45
Alpha helix (%)	20.98	22.28	20.47	22.28	20.47	17.90	21.47	21.47	23.56	22.77
Beta turn (%)	2.59	2.85	2.33	2.85	2.33	2.78	2.88	2.88	2.88	2.88
Extended strand (%)	17.36	17.62	18.13	17.62	18.13	20.99	18.59	18.59	15.71	19.90

Table S4 The coverage and consistency between the fragment sequence of qPCR obtained in this study and the TP53 sequence in the NCBI database

Species	Coverage (%)	Identity (%)
Buffalo_X1	100	98.94
Buffalo_X2	100	100
Buffalo_X3	100	100
Cattle	100	98.40
Yak	100	98.94
Zebu	100	98.40
Bison_X1	100	98.94
Bison_X2	100	98.25
Goat_X1	100	94.76
Goat_X2	100	94.76
Sheep_X1	100	93.72
Sheep_X2	100	93.72

Table S5 Relative synonymous codon usage values of *TP53* gene in buffalo

Amino acid	Codon	B1	B2	B3
Phe	UUU	0.91	0.91	0.91
	UUC	1.09	1.09	1.09
Leu	UUA	0.16	0.16	0.16
	UUG	0.65	0.65	0.65
	CUU	0.97	0.97	0.97
	CUC	0.81	0.81	0.81
	CUA	0.32	0.32	0.32
	CUG	3.08	3.08	3.08
Ile	AUU	0	0	0
	AUC	3	3	3
	AUA	0	0	0
Met	AUG	1	1	1
Val	GUU	0.71	0.71	0.71
	GUC	0.71	0.71	0.71
	GUA	0	0	0
	GUG	2.59	2.59	2.59
Ser	UCU	1.74	1.74	1.74
	UCC	2.21	2.21	2.21
	UCA	0.32	0.32	0.47
	UCG	0.32	0.32	0.16
Pro	CCU	1.14	1.14	1.24
	CCC	1.33	1.33	1.24
	CCA	1.14	1.14	1.14
	CCG	0.38	0.38	0.38
Thr	ACU	0.55	0.55	0.55
	ACC	2.36	2.36	2.36
	ACA	0.73	0.73	0.73
	ACG	0.36	0.36	0.36
Ala	GCU	0.6	0.8	0.8
	GCC	2.4	2.2	2.2
	GCA	1	1	1
	GCG	0	0	0
Tyr	UAU	1.09	1.09	1.09
	UAC	0.91	0.91	0.91
TER	UAA	0	0	0
	UAG	0	0	0
His	CAU	0.8	0.8	0.8
	CAC	1.2	1.2	1.2
Gln	CAA	0.36	0.36	0.36
	CAG	1.64	1.64	1.64
Asn	AAU	0.82	0.82	0.82
	AAC	1.18	1.18	1.18
Lys	AAA	0.4	0.4	0.4
	AAG	1.6	1.6	1.6
Asp	GAU	0.84	0.84	0.84
	GAC	1.16	1.16	1.16
Glu	GAA	0.81	0.81	0.81
	GAG	1.19	1.19	1.19
Cys	UGU	1.23	1.23	1.23
	UGC	0.77	0.77	0.77
TER	UGA	3	3	3
Trp	UGG	1	1	1
Arg	CGU	0.67	0.67	0.67
	CGC	1.78	1.78	1.78
	CGA	0.89	0.89	0.89
	CGG	1.11	1.11	1.11
Ser	AGU	0.32	0.32	0.32
	AGC	1.11	1.11	1.11
Arg	AGA	0.67	0.67	0.67
	AGG	0.89	0.89	0.89
Gly	GGU	0.67	0.67	0.67
	GGC	0.67	0.67	0.67
	GGA	0.67	0.67	0.67
	GGG	2	2	2

Table S6 Information for haplotypes

Haplotype	Accession number of nucleotide sequences
B1	OL456217
B2	XM_006062871
B3	NM_001290844
Cattle_hap1	DQ656491
Cattle_hap2	NM_174201
Cattle_hap3	NC_037346
Cattle_hap4	AB571118
Cattle_hap5	X81704
Cattle_hap6	XM_027519387
Cattle_hap7	XM_027519388
Cattle_hap8	D49825
Yak_hap1	XM_005894802
Yak_hap2	NW_005393612
Yak_hap3	KR185965
Zebu_hap1	XM_019982111
Zebu_hap2	NC_032668
Bison_hap1	XM_010843072
Goat_hap1	XM_018064593
Sheep_hap1	FJ855223
Sheep_hap2	NM_001009403
